# Micro- and Nanoplastic Pollution and Renal Health: Human Evidence, Mechanisms, and Clinical Implications

**DOI:** 10.3390/jox16040149

**Published:** 2026-08-13

**Authors:** Aris Tsalouchos, Pietro Claudio Dattolo

**Affiliations:** Nephrology and Dialysis Unit Firenze 2, Department of Medical Specialties, Azienda USL Toscana Centro, Santa Maria Annunziata Hospital, 50012 Florence, Italy; pietroclaudio.dattolo@uslcentro.toscana.it

**Keywords:** microplastics, nanoplastics, kidney, nephrotoxicity, chronic kidney disease, biomonitoring, dialysis, xenobiotics

## Abstract

Micro- and nanoplastics (MNPs) are reported in blood, urine, and tissues, including the kidney, raising concern that the urinary system may be both a target and a route of elimination. This narrative review integrates analytical, toxicokinetic, experimental, epidemiological, and dialysis-related evidence on MNPs and renal health. Human studies report polymer-confirmed particles or polymer-associated signals in blood, urine, and kidney-related specimens, supporting systemic presence and compatibility with renal exposure; however, estimates are strongly method-dependent and do not establish anatomical localization, tissue dose, clearance, or a disease threshold. Cell, kidney-organoid, and animal studies consistently identify oxidative and mitochondrial stress, endoplasmic-reticulum dysfunction, inflammation, altered autophagy, regulated cell death, senescence, and fibrotic remodelling. Effects vary with polymer, size, shape, surface ageing, route, dose, and chemical co-exposures, and many experiments remain difficult to map to typical human exposure. Direct human outcome evidence is limited to cross-sectional or exploratory studies; plasticizer epidemiology is informative for chemical co-exposure but cannot establish particle-specific toxicity. Patients with chronic kidney disease may be more susceptible, while kidney dysfunction may also alter measured blood or urinary concentrations. Dialysis introduces an additional, incompletely quantified exposure system. MNP-related renal injury is biologically plausible, but causal contribution to human kidney disease and clinical actionability remain unproven. Standardized methods, paired-matrix kinetics, prospective cohorts, and complete dialysis mass-balance studies are priorities.

## 1. Introduction

Plastic production and use have created a persistent continuum of debris ranging from visible fragments to particles below the resolution of routine microscopy. Microplastics (MPs) are commonly defined as plastic particles smaller than 5 mm, whereas the boundary assigned to nanoplastics (NPs) varies among disciplines and is often operational rather than biological [1,2]. This terminology matters because a millimetre-scale fibre, a 5-µm fragment, and a 50-nm particle differ markedly in surface-area-to-mass ratio, aggregation, protein-corona formation, cellular uptake, and ability to cross biological barriers. The term micro- and nanoplastics (MNPs) is therefore convenient for describing a pollution continuum, but it should not imply that all particles share a common toxicokinetic profile or renal hazard.

Human exposure occurs through ingestion, inhalation, and contact with consumer or medical products. Plastics also contain additives and residual monomers and can adsorb other environmental contaminants; thus, the biologically encountered material may comprise the particle, its surface corona, leachable chemicals, and co-pollutants. The public-health implications of this mixture extend from the full plastics life cycle to individual exposure [3]. Analytical studies have reported plastic-associated signals in human blood [4], placenta [5], lung [6], kidney and urine [7], atherosclerotic plaque [8], and post-mortem organs including brain, liver, and kidney [9]. These observations support internal exposure. They do not, by themselves, establish that the detected material caused tissue injury or disease, and several high-profile measurements remain challenging to compare because the reported measurand may be particle number, polymer mass, percentage of positive samples, or a spectroscopic match.

The kidney is a plausible target for circulating MNPs and their chemical cargo. Renal blood flow creates extensive contact with the glomerular microvasculature; filtration and tubular transport concentrate many xenobiotics; proximal tubular cells have high energetic requirements; and resident and recruited immune cells can amplify injury. At the same time, the glomerular filtration barrier is highly selective, and the renal fate of a particle cannot be inferred from nominal diameter alone. Effective size and surface properties change through aggregation, ageing, and protein adsorption. A particle detected after digestion of kidney tissue may have been intravascular, trapped in a glomerulus, internalized by tubular or immune cells, retained in the interstitium, or introduced during collection and processing. Similarly, a urinary signal may reflect filtration, secretion, epithelial shedding, downstream urinary-tract sources, or contamination.

Interest in renal effects has expanded rapidly. Recent reviews summarize kidney and cardiovascular mechanisms [10] and earlier evidence for MNP deposition and nephrotoxicity [11]. Since those syntheses, the field has added multimodal human tissue studies, urinary biomonitoring, kidney organoids, more realistic particle geometries, models of pre-existing renal disease, and the first patient-level investigations of dialysis-associated exposure. The central question has consequently shifted from whether a plastic-associated signal can be found to whether the available evidence supports a clinically relevant contribution to kidney disease.

This narrative review evaluates that question across five linked domains: human exposure and evidence relevant to renal exposure; analytical validity; renal handling; experimental injury and mechanism; and human clinical relevance, including chronic kidney disease (CKD), development, transplantation, nephrolithiasis, renal cancer, and dialysis. Dialysis is treated as a separate major component because repeated, high-volume contact with polymer-rich extracorporeal or intraperitoneal systems creates a distinctive and clinically important source-and-sink problem that can be resolved only through complete mass-balance designs. PubMed was searched through 26 July 2026 using the following core query: (microplastic*[Title/Abstract] OR nanoplastic*[Title/Abstract] OR “micro- and nanoplastics”[Title/Abstract]) AND (kidney[Title/Abstract] OR renal[Title/Abstract] OR urine[Title/Abstract] OR urinary[Title/Abstract] OR glomerul*[Title/Abstract] OR tubul*[Title/Abstract] OR nephro*[Title/Abstract] OR dialysis[Title/Abstract] OR hemodialysis[Title/Abstract] OR haemodialysis[Title/Abstract] OR “peritoneal dialysis”[Title/Abstract]).

The core search was supplemented by targeted combinations of MNP terms with individual analytical techniques, polymers, mechanisms, and renal conditions, together with backward citation searching of relevant reviews and primary reports. English-language, peer-reviewed primary human, animal, kidney-organoid, and renal-cell studies were prioritized when they used direct particle or polymer measurements, defined MNP exposures, renal endpoints, analytical controls, or mechanistic interventions. Methodological and review articles were retained for definitions and critical context. Ecological reports without renal endpoints and studies of plastic-associated chemicals alone were not treated as direct evidence of particle toxicity. Because study designs, particles, matrices, size windows, and outcome measures were highly heterogeneous, the evidence was synthesized narratively rather than pooled quantitatively.

## 2. Human Exposure, Systemic Distribution, and Evidence Relevant to Renal Exposure

### 2.1. From Environmental Exposure to the Circulation

Demonstrating systemic entry is a prerequisite for haematogenous delivery to the kidney. In a pioneering study of 22 healthy volunteers, double-shot pyrolysis gas chromatography–mass spectrometry (Py-GC/MS) quantified plastic polymers of at least approximately 700 nm in blood, with a mean total concentration of 1.6 µg/mL [4]. Other studies identified micrometre-scale particles in placenta and lung using Raman or Fourier-transform infrared (FTIR) microspectroscopy [5,6]. These reports are consistent with uptake after ingestion or inhalation, but their outputs are not interchangeable: thermal analysis quantifies polymer mass after destruction of the sample, whereas microspectroscopy counts individual particles within an instrument-specific size window.

Evidence from tissues supports distribution beyond the portal and pulmonary interfaces. In carotid endarterectomy specimens, polyethylene was detected by Py-GC/MS in 58.4% of plaques and polyvinyl chloride in 12.1%; the presence of plastic-associated material was associated with a higher composite risk of myocardial infarction, stroke, or death during follow-up [8]. This study is important because it linked a tissue measurement with longitudinal outcomes, but it concerned a selected cardiovascular population and does not define renal risk. Post-mortem analyses using complementary thermal, spectroscopic, and electron-microscopic methods have also reported MNPs in kidney, liver, and brain, with pronounced variation among tissues and sampling periods [9,12]. Autopsy studies broaden the biodistribution map but remain vulnerable to post-mortem sampling, digestion, recovery, and contamination effects and generally cannot determine temporality.

Animal and modelling studies indicate that systemic delivery is strongly size- and route-dependent. After a single exposure, fluorescently labelled particles produced time-dependent blood and urinary signals in rodents, although fluorescence and electron microscopy did not always provide concordant evidence for intact particles [13]. A physiologically based toxicokinetic model predicted substantial size dependence around the submicrometre range and emphasized gastrointestinal absorption and faecal excretion as determinants of internal dose [14]. More recent mammalian tracing experiments likewise found that translocation across biological barriers varied with size [15]. These findings make renal access plausible but do not support a universal filtration threshold or conversion from an administered dose to a human renal dose.

### 2.2. Direct Evidence in Human Kidney and Urinary-System Specimens

Massardo and colleagues provided the first polymer-confirmed evidence in human kidney tissue and urine using micro-Raman spectroscopy [7]. Healthy portions of ten nephrectomy kidneys and ten urine samples from healthy donors were analysed. The study identified 26 particles across kidney and urine specimens, with sizes of 1–29 µm in kidney and 3–13 µm in urine; polyethylene and polystyrene were the most frequent polymers. Extensive procedural, reagent, water, and filter controls were a major strength. However, complete digestion removed anatomical localization, the kidney and urine samples were not paired for mass-balance analysis, and no histological or functional phenotype was linked to the particles. The result therefore establishes the presence of polymer-confirmed particles in renal specimens, not parenchymal access, accumulation, filtration, toxicity, or disease causation.

Subsequent studies have extended detection to clinically obtained renal-system tissues. Multimodal analysis of tissue from 28 kidney donors reported polymer signals in kidney, renal vessels, ureter, and adrenal gland [16]. Exploratory correlations with recipients’ blood pressure 15 days after transplantation were mostly non-significant after correction for multiple testing, and the measured particles—many reported between 20 and 500 µm—raise unresolved questions about tissue localization and the relation between particle counts and polymer mass. In paired clear-cell renal cell carcinoma (ccRCC) and adjacent tissue, laser direct infrared (LD-IR) spectroscopy, scanning electron microscopy, and Py-GC/MS showed differences in polymer composition and higher total signals in tumour specimens [17]. Detection in tumour tissue is not evidence that MNPs initiated cancer: altered vascularity, tissue architecture, inflammation, and sampling may all affect retention or measurement.

Kidney stones provide another long-lived matrix in which material may be incorporated over time. A 2026 multimodal study reported six polymer types in all examined stones, with a median polymer mass of 12.89 µg/g and predominantly irregular particles in the 20–100 µm range; accompanying multi-omics experiments linked chronic low-dose exposure to mitochondrial and inflammatory responses in renal cells [18]. The finding is hypothesis-generating for nephrolithiasis, but a stone-associated particle may be a nidus, a passenger incorporated during growth, or an external contaminant. Demonstrating a lithogenic role requires prospective exposure data and comparison with rigorously processed stone-free controls.

### 2.3. Urinary Biomonitoring: An Attractive but Unresolved Matrix

Urine is non-invasive and clinically familiar, which makes it an appealing exposure matrix. Early Raman-based work reported microplastics in the urine of a small number of adults [19]. Later studies found regional differences in urinary MNP signals [20], documented the importance of collection and sampling conditions [21], and showed that the apparent particle profile changes when smaller size fractions are interrogated [22]. Double-shot Py-GC/MS with internal-standard calibration has extended mass-based detection toward particles reported at or above 300 nm [23]. Cascaded microfiltration followed by Py-GC/MS achieved matrix-matched quantification of six polymers and showed that a 0.7–1.2 µm operational fraction accounted for much of the measured mass in multiple biological matrices [24].

Two 2026 studies further illustrate both the potential and the uncertainty of urinary biomonitoring. Paired blood and urine measurements in 98 environmentally exposed volunteers detected at least one of ten polymers in every participant, with higher overall detection in urine than blood and polymer-specific partitioning patterns [25]. In 29 schoolchildren, MNPs below 10 µm were reported in every first-morning urine sample using scanning electron microscopy/energy-dispersive X-ray analysis with Py-GC/MS polymer characterization [26]. These studies suggest that urine contains structured exposure information, but neither validates a clearance model. A concentration in a spot sample can vary with urine flow, recent exposure, collection materials, lower size limit, blank subtraction, and normalization. Larger particles also challenge simple assumptions of glomerular filtration.

**Figure 1:**
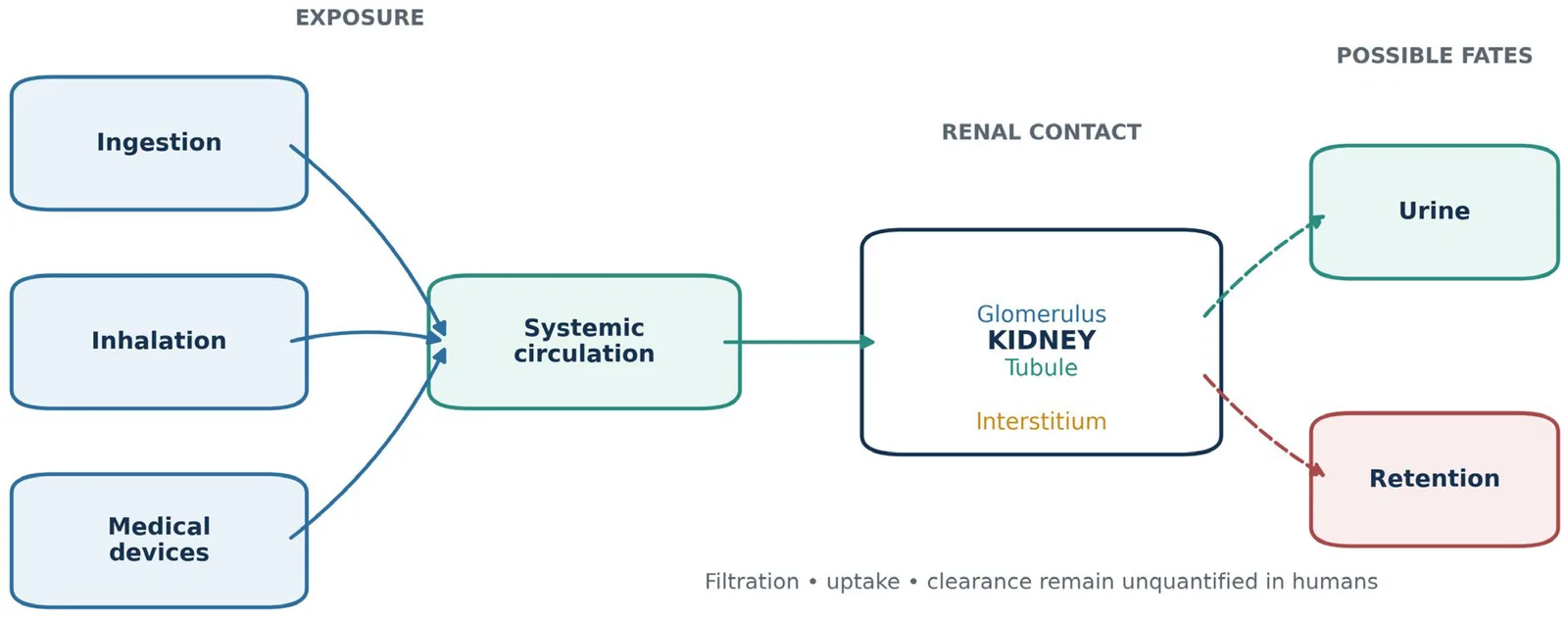
Exposure pathways and renal disposition of micro- and nanoplastics. Ingestion and inhalation are established environmental routes, whereas medical devices can add healthcare-associated exposure. Systemically available material may remain in blood, contact renal vascular and epithelial compartments, be retained, or appear in urine. The relative contributions of glomerular passage, tubular handling, urinary-tract shedding, and contamination remain unresolved; arrows indicate plausible pathways rather than quantified human fluxes.

Urinary MNPs should therefore not yet be described as a biomarker of “kidney burden.” A rigorous renal-handling study would require repeated paired blood and timed urine sampling, measurement of urine flow and filtration, size-resolved polymer confirmation in both matrices, full procedural blanks, recovery experiments, and ideally tissue or imaging information. Until such data exist, a urinary signal may indicate exposure or excretion but does not distinguish filtration, tubular release, urinary-tract shedding, or contamination. These exposure pathways and unresolved aspects of renal disposition are summarized in Figure 1.

## 3. Analytical Challenges in Renal Matrices

MNP analysis is not a single assay but a chain of collection, digestion, separation, identification, and quantification steps. Reproducible reporting requires particle characterization, quality assurance and quality control, field and laboratory blanks, recovery data, matrix effects, and explicit treatment of values near detection limits [27,28]. These requirements are particularly demanding in human studies, where the expected signal is low, tissue is chemically complex, and plastics are ubiquitous in the clinical and laboratory environment.

Spectroscopic and thermal methods answer different questions. Raman and Fourier-transform infrared (FTIR) microspectroscopy retain particle morphology, but their practical lower size limits depend on the matrix, substrate, optics, and classification rules. Raman can generally interrogate smaller particles than FTIR, yet fluorescence, laser-induced damage, weak scattering, long acquisition times, weathering, biological residues, and spectral-library dependence restrict sensitivity and throughput. FTIR and laser direct infrared (LD-IR) imaging enable faster mapping and automated classification, but diffraction, particle thickness, substrate compatibility, and match-threshold algorithms reduce sensitivity for the smallest fractions. Routine micro-Raman, micro-FTIR, and LD-IR measurements should not therefore be assumed to directly characterize true nanoplastics [29,30].

Py-GC/MS can quantify low polymer masses and detect polymers that particle-counting methods miss, but it destroys size, shape, surface, and anatomical information and may include dissolved or non-particulate polymer-associated material unless separation is operationally defined. This distinction is especially important for nanoplastics: filtration or ultracentrifugation followed by thermal analysis quantifies polymer mass in an operational size fraction, not necessarily intact nanoparticles, whereas electron microscopy can visualize nanoscale morphology but usually cannot establish polymer identity alone. Field-flow fractionation, light-scattering, microscopy, spectroscopy, and thermal analysis each cover only part of the problem. Credible nanoplastic claims therefore require size-fractionation controls, recovery across the claimed range, contamination control, and orthogonal confirmation, with explicit reporting of whether the measurand is particle number, polymer mass, morphology, or surface-associated signal [29,30,31,32,33,34].

Cross-platform evidence confirms these method-dependent contrasts. In paired endometrial and urine samples, Raman microscopy characterized individual particles of approximately 1.2–7 µm, whereas Py-GC/MS identified additional polymer co-occurrences in samples initially classified as single-polymer by Raman [31]. A multistep digestion protocol for human tissue improved organic-matrix removal while preserving particles for micro-Raman analysis, illustrating that digestion chemistry must be validated for the target polymers and matrix [32]. No method currently captures particle number, polymer mass, shape, nanoscale fraction, surface chemistry, and anatomical localization simultaneously.

Contamination control is not optional. Renal surgery, tissue storage, urinary collection, dialysis tubing, laboratory clothing, filters, and airborne fibres are all potential sources. Procedural blanks should accompany every batch; collection containers and instruments should be polymer-audited; recovery should be assessed across relevant particle sizes and polymers; and both raw and blank-corrected results should be reported. Fluorescent tracing requires additional controls because dyes can leach from labelled particles and accumulate independently, while biological autofluorescence can create false-positive localization [33].

The newest systematic assessment of methods in human biological materials still finds marked heterogeneity and limited validation across matrices [34]. Consequently, agreement within one platform is not equivalent to external validity. Orthogonal confirmation is especially important when an unexpected tissue concentration, very small particle, or unusually large urinary particle drives the biological conclusion. The principal analytical approaches and their interpretative limitations are summarized in Table 1.

## 4. Renal Handling and Toxicokinetics

The renal fate of MNPs depends on their state in biological fluids rather than their nominal diameter in a stock suspension. Protein adsorption can alter charge, aggregation, macrophage recognition, and effective hydrodynamic size. Weathering and degradation change surface oxidation and the release of additives or oligomers. Shape also influences flow and tissue contact. A rigid spherical bead is therefore an imperfect surrogate for an irregular, aged environmental fragment.

The glomerular filtration barrier comprises fenestrated endothelium, glomerular basement membrane, and podocyte slit diaphragms. It is not a passive pore-size filter. Charge, deformability, protein binding, haemodynamics, and barrier integrity all influence passage. Larger circulating particles may remain intravascular or be taken up by endothelial and phagocytic cells, whereas sufficiently small or deformable particles may enter the filtrate. Peritubular delivery and basolateral uptake provide potential routes that do not require glomerular filtration. Once in tubular cells, particles may undergo lysosomal sequestration, exocytosis, epithelial shedding, or prolonged retention.

Experimental kinetics support rapid faecal elimination of most ingested material and much smaller systemic fractions [13,14,15]. A 2026 study comparing realistic irregular fragments with spherical particles reported that more than 99% of the administered material was excreted in faeces, yet irregular fragments persisted longer in circulation, appeared in glomeruli and tubules, attracted phagocytic cells, and produced albumin hyperfiltration and local lesions; comparable effects were not observed with spheres [35]. Although this work improves shape realism, administered dose, labelling, and species differences still limit direct extrapolation.

Renal excretion should be distinguished from renal clearance. Detection in urine establishes that material or a polymer-associated signal reached the sample; clearance requires a validated relation between plasma concentration, urine concentration, flow, and time. Tissue concentration likewise requires a defined denominator and knowledge of whether the signal was intravascular or parenchymal. Human studies have not yet provided a complete blood–kidney–urine mass balance. This gap is central to causal interpretation because CKD could increase circulating concentrations by reducing elimination, decrease urinary concentrations by lowering filtration, increase urinary particles through barrier injury or epithelial shedding, or alter the biological response to an unchanged exposure.

## 5. Experimental Evidence of Renal Injury

### 5.1. Renal Cells and Kidney Organoids

Proximal tubular cells are the most frequently studied human renal model. In HK-2 cells, polystyrene microplastics increased mitochondrial reactive oxygen species (ROS), endoplasmic-reticulum (ER) stress, inflammatory signals, and autophagy; the mitochondrial antioxidant MitoTEMPO attenuated several of these responses [36]. Polystyrene nanoplastics induced size- and dose-dependent apoptosis through oxidative stress and p38/ERK mitogen-activated protein kinase signalling [37]. A cross-polymer comparison subsequently showed that uptake and short-term toxicity were not determined by size alone: polyethylene and poly(methyl methacrylate) nanoparticles produced greater morphological disruption than several polystyrene preparations, whereas low concentrations had minimal effects and marked viability loss occurred mainly at 100–200 µg/mL [38]. These studies demonstrate hazard and property dependence, but direct immersion of an immortalized monolayer does not reproduce the delivered dose, corona, flow, or clearance of the human proximal tubule.

Real-world exposure is also a mixture problem. In human proximal tubular epithelial cells, combined polyethylene microplastic and bisphenol A exposure reduced viability and increased oxidative and inflammatory responses more than either exposure alone [39]. Similar enhancement has been reported with metals and other adsorbed chemicals in experimental systems. Such findings show that particle–chemical interactions can modify toxicity, but they do not justify attributing the combined effect solely to the physical particle.

Human pluripotent-stem-cell-derived kidney organoids add multicellular architecture and developmental patterning. Exposure to 1-µm polystyrene particles during the nephron-progenitor stage reduced organoid size, increased ROS and apoptosis, and disturbed proximal and distal tubular patterning through altered Notch signalling [40]. Follow-up work linked growth retardation and abnormal tubules to mitochondrial ROS, DNA damage, reduced glycolysis, and compensatory tricarboxylic-acid-cycle activity [41]. Other studies identified DDIT4-mediated mTOR inhibition, autophagy, and apoptosis [42] and activation of the BCL-2/Bax/caspase pathway [43]. Silencing DDIT4 attenuated autophagy and apoptosis, strengthening the causal role of that pathway within the model [42].

Organoids are relevant to developmental susceptibility, but they remain immature and lack mature filtration, perfusion, immune-cell trafficking, and urinary excretion. Moreover, several studies selected nominal concentrations by reference to polymer mass reported in whole blood, even though a blood mass concentration does not establish the concentration of intact particles reaching nephron progenitors. An inhalation study that combined mice, HK-2 cells, and kidney organoids found greater sensitivity in organoids than in the immortalized cell line [44], again suggesting that model choice materially affects the apparent hazard.

### 5.2. Whole-Kidney Injury in Animal Models

Animal models integrate absorption, circulation, immune responses, and renal physiology. Oral exposure to polystyrene particles of 50 nm to 4 µm produced size-dependent aggregation in the gastrointestinal tract, renal signals, oxidative stress, inflammation, altered biochemical markers, and histological damage in mice [45]. In combined in-vitro and in-vivo work, polystyrene exposure produced tubular stress and renal histopathology consistent with the cell responses described above [36]. These studies established early proof of renal hazard but used pristine particles and exposure regimens designed for effect detection.

More recent experiments have moved toward organ crosstalk and pathway perturbation. Polystyrene microplastics impaired the intestinal barrier and activated the renal complement C5a/C5aR pathway; an antibiotic intervention and the C5aR inhibitor PMX53 attenuated injury, supporting a gut–kidney component [46]. Co-exposure with di(2-ethylhexyl) phthalate intensified oxidative stress, NF-κB/NLRP3 activation, pyroptosis, and inflammation; N-acetylcysteine and the NLRP3 inhibitor MCC950 reduced several effects [47]. These interventions increase mechanistic confidence, although antibiotics, antioxidants, and signalling inhibitors can have broad actions and do not prove that a single pathway is exclusive.

Several models describe progression from tubular stress to remodelling. Six months of polystyrene exposure induced inflammation, ferroptosis, and renal fibrosis; ferrostatin-1 attenuated epithelial ferroptosis and fibroblast activation [48]. A separate model linked fibrosis to Klotho/Wnt/β-catenin-dependent tubular senescence and showed improvement after restoration of Klotho function [49]. Nanoplastic-induced lysosomal dysfunction impaired autophagic flux, whereas lysosomal exocytosis contributed to particle clearance and recovery after exposure [50]. Ultraviolet ageing enhanced polystyrene nanoplastic toxicity by altering transferrin interactions, intracellular iron, lipid peroxidation, and ferroptosis [51]. Together, these studies indicate that persistence, surface transformation, and failure of cellular clearance can convert early stress into maladaptive repair.

Renal dysfunction has also been assessed at the organ level. After 12 weeks of polyethylene exposure, radionuclide imaging with ^99m^Tc-DMSA and ^99m^Tc-DTPA, serum and urinary markers, and histology indicated impaired renal function in mice [52]. In Wistar rats, oral polyethylene microplastics increased serum creatinine, urea, cystatin C, albumin-to-creatinine ratio, and kidney injury molecule-1 in a dose-dependent manner while suppressing mitochondrial respiratory complexes and producing glomerular and tubular lesions [53]. The inclusion of polyethylene helps counter the field’s strong polystyrene bias, but daily doses of 15 and 60 mg/kg remain far above any established human renal dose.

Newer studies refine the identity of the affected compartment and the importance of particle properties. Chronic nanoplastic exposure produced tubular-specific injury, mitochondrial oxidative-phosphorylation failure, STING-dependent inflammation, senescence, and fibrosis; activation of PGC1α improved mitochondrial function and attenuated these outcomes [54]. Irregular fragments, but not matched spheres, disrupted glomerular capillary flow and barrier integrity [35]. Polystyrene microplastics activated non-canonical TGF-β signalling, epithelial–mesenchymal transition, metabolic reprogramming, and early fibrosis [55]. Partially degraded polylactic-acid particles were taken up by renal macrophages through MSR1 and produced greater inflammatory injury than higher-molecular-weight polymer particles; inhibition of MSR1 or PI3K/AKT narrowed the toxicity difference [56]. These results argue against treating “microplastic exposure” as a uniform dose.

The urinary system may also be vulnerable to disease-specific interactions. In a calcium-oxalate nephrolithiasis model, polystyrene nanoplastics changed crystal morphology, increased crystal adhesion to tubular cells, promoted macrophage recruitment and ferroptosis, and aggravated stone formation; polymers were also reported in human stones [57]. The human observation and experimental mechanism are mutually informative, but neither establishes that ordinary exposure initiates stones in the population. Representative experimental models and their principal translational limitations are summarized in Table 2.

## 6. Mechanisms of MNP-Associated Renal Toxicity

### 6.1. Oxidative Stress and Mitochondrial Dysfunction

Oxidative stress is the most reproducible signal across renal models, but it should be interpreted as a common response rather than a particle-specific mechanism. MNP contact, internalization, lysosomal stress, surface redox activity, or leached chemicals can increase ROS. Proximal tubular cells are especially vulnerable because they depend on mitochondrial oxidative phosphorylation. Consequences reported across studies include loss of membrane potential, reduced ATP production, mitochondrial structural damage, lipid peroxidation, DNA damage, and activation of stress kinases [36,37,38,39,41,44,53,54].

Mechanistic depth is strongest where mitochondrial injury precedes a renal phenotype and targeted intervention provides rescue. MitoTEMPO attenuated mitochondrial ROS and downstream ER-stress and autophagy signals [36]. PGC1α activation restored mitochondrial function and reduced inflammation, senescence, and fibrotic remodelling in cells and mice [54]. In organoids, altered glycolytic and tricarboxylic-acid-cycle flux accompanied impaired nephron growth [41]. These studies support mitochondrial dysfunction as a central event within several models, while leaving open whether equivalent intracellular doses occur in humans.

### 6.2. ER Stress, Autophagy, and Lysosomal Handling

The ER–autophagy–lysosome axis is repeatedly disturbed after MNP exposure. ER-stress proteins, LC3, and Beclin 1 increase in tubular models [36]. DDIT4-mediated mTOR inhibition linked exposure to autophagy and apoptosis in kidney organoids [42]. Nanoplastics can promote autophagosome formation while impairing lysosomal degradation, leading to blocked autophagic flux and fibrosis; recovery may depend on lysosomal exocytosis and particle clearance [50]. This dual role is biologically plausible because lysosomes are both the destination for internalized particles and a determinant of whether damaged proteins, organelles, and particles can be removed.

Autophagy markers alone do not reveal whether flux is increased or blocked. Future experiments should combine time-resolved flux assays, lysosomal function, intracellular particle localization, and recovery after exposure. Without those elements, increased LC3 may reflect protective recycling, impaired degradation, or both.

### 6.3. Inflammation, Innate Immunity, and the Gut–Kidney Axis

MNPs can activate inflammatory signalling after direct renal contact or through extra-renal pathways. Reported mechanisms include NF-κB, NLRP3 inflammasome activation, complement C5a/C5aR, STING signalling after mitochondrial injury, chemokine-mediated macrophage recruitment, and extracellular-vesicle communication. The gut–kidney study provides intervention-supported evidence that intestinal barrier disruption and complement activation contribute to renal injury [46]. Co-exposure with DEHP activated NF-κB/NLRP3 and pyroptosis [47]. Degraded polylactic-acid particles were decoded by macrophage scavenger receptor 1, generating a CCL2-dependent inflammatory loop [56].

These pathways converge with established mechanisms of AKI and CKD and may therefore amplify rather than uniquely initiate disease. This is particularly relevant in susceptible kidneys, where basal inflammation, oxidative stress, endothelial dysfunction, and impaired repair are already present. It also means that generic cytokine changes cannot identify an MNP-specific signature.

### 6.4. Regulated Cell Death, Senescence, and Fibrosis

Apoptosis, pyroptosis, and ferroptosis appear in different experimental contexts. Oxidative and ER stress can activate BCL-2-family and caspase pathways [37,43]. NLRP3/caspase-1 and gasdermin-dependent pyroptosis has been described in co-exposure and kidney-injury models [47,58]. Iron dysregulation and lipid peroxidation support ferroptosis in chronic, aged-particle, and stone models [48,51,57]. These processes can release inflammatory signals, promote fibroblast activation, and impair tubular repair.

Senescence supplies a plausible bridge from repeated sublethal stress to chronic remodelling. Klotho/Wnt/β-catenin-dependent tubular senescence increased TGF-β1 signalling and fibroblast activation [49], while persistent mitochondrial dysfunction induced senescence and fibrosis through a partially separable pathway from STING-mediated inflammation [54]. Non-canonical TGF-β signalling and epithelial phenotypic change have also been observed [55]. Although the term epithelial–mesenchymal transition should be used cautiously in vivo, the combined evidence supports maladaptive tubular–interstitial crosstalk as an experimental outcome.

### 6.5. Particle Properties and Chemical Co-Exposure

Toxicity varies with polymer chemistry, size, shape, surface charge, ageing, aggregation, and degradation state [35,38,45,51,56]. Smaller particles may have greater cellular access, but aggregation can make an intermediate nominal size more biologically active [45]. Irregular fragments behave differently from spheres [35]. Ultraviolet ageing can increase oxidative surface chemistry and change protein binding [51]. Biodegradable plastic is not necessarily biologically inert: oligomeric polylactic-acid material produced a different renal immune response from the parent polymer [56].

**Figure 2:**
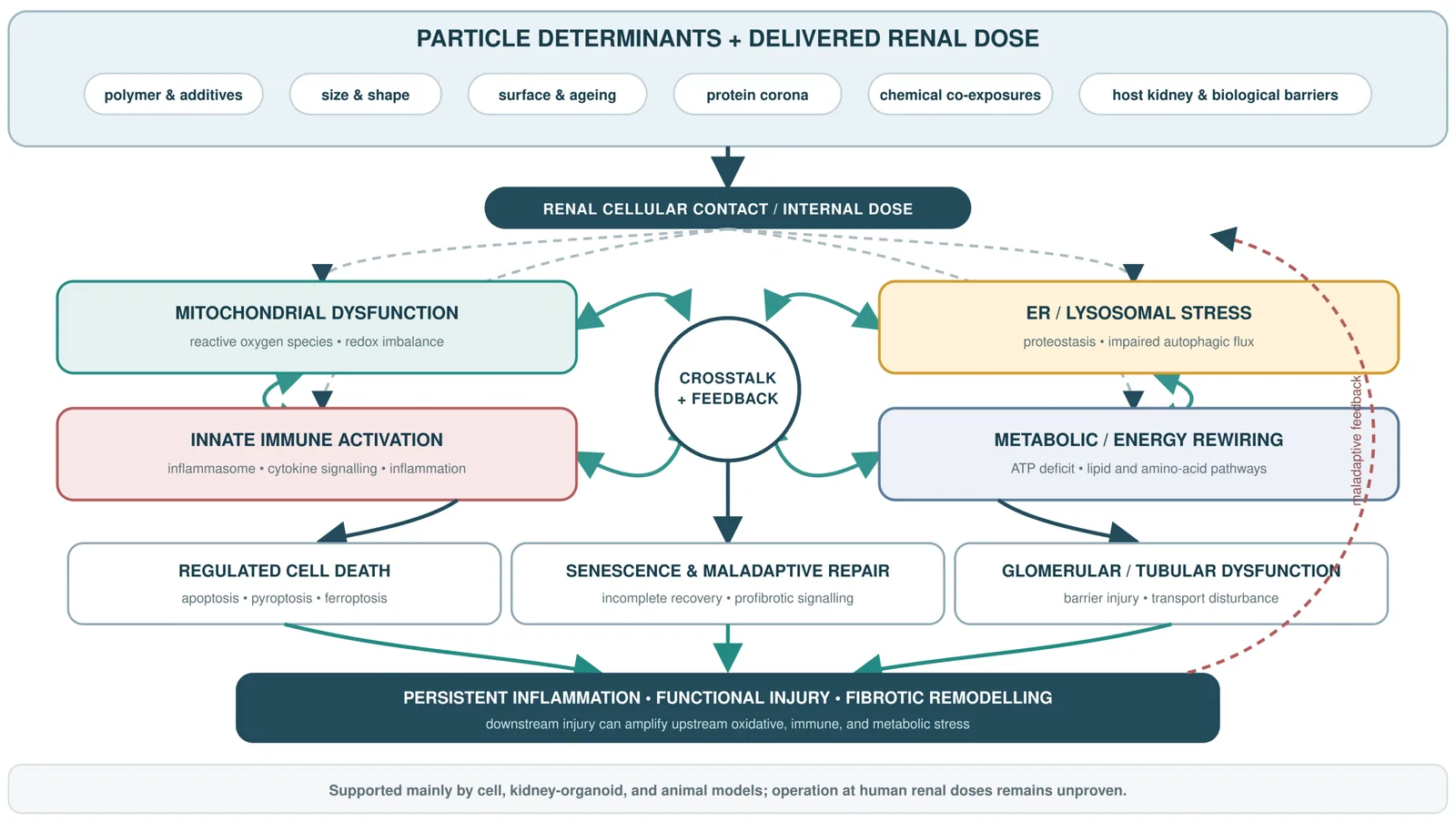
Interconnected mechanisms proposed for MNP-associated renal injury. Particle properties, chemical cargo, biological barriers, and delivered renal dose influence cellular contact. Mitochondrial and oxidative stress, ER/lysosomal dysfunction and impaired autophagic flux, innate immune activation, and metabolic rewiring interact through bidirectional crosstalk and feedback. Their convergence can promote regulated cell death, senescence, maladaptive repair, glomerular or tubular dysfunction, persistent inflammation, and fibrotic remodelling. These pathways are supported primarily by experimental models; their operation at human renal doses has not been established.

Particles also transport or co-occur with plasticizers, metals, persistent organic pollutants, and other xenobiotics. Experiments with BPA or DEHP demonstrate enhanced renal injury under combined exposure [39,47]. Accordingly, three distinct questions should be kept separate: the toxicity of the physical particle, the toxicity of particle-associated chemicals, and interaction between them. Real-world risk may reflect all three, but a study measuring only a urinary chemical metabolite does not measure intact MNP exposure. The interacting pathways proposed for MNP-associated renal injury are summarized in Figure 2.

## 7. Human Evidence for Renal Outcomes

### 7.1. Direct Particle Measurements and Renal Phenotypes

Human outcome evidence remains much smaller than the detection and experimental literatures. In 50 healthy adults from Barcelona, six polymers between approximately 0.7 and 20 µm were measured in stool and spot urine by high-performance liquid chromatography coupled to high-resolution mass spectrometry [59]. At least one urinary polymer was detected in 48% of participants. Presence of any urinary polymer was associated with a higher urinary albumin-to-creatinine ratio (ratio of means 2.18; 95% confidence interval 1.17–4.01). However, all albumin-to-creatinine ratios remained below 30 mg/g, the design was cross-sectional, individual polymer detections were sparse, and the associations did not remain significant after false-discovery-rate correction. The study provides a preliminary renal signal, not evidence of albuminuric CKD.

A Chinese birth cohort of 1350 pregnancies reported microplastics in placental samples and positive mixture associations—driven mainly by polyvinyl chloride—with cord-blood creatinine and cystatin C [60]. The sample size and mixture modelling are notable, but placental counts are a proxy for fetal exposure, cord-blood biomarkers have gestational and perinatal determinants, and the study cannot establish postnatal renal dysfunction. Replication with standardized placental analysis, neonatal measured or longitudinal estimated GFR, and childhood follow-up is needed.

The transplant-donor study found widespread polymer signals across donor kidney-associated tissues but no robust relation with early recipient blood pressure after multiple-testing correction [16]. ccRCC studies observed more polymer-associated material in tumour than adjacent tissue and linked experimental polystyrene exposure to NF-κB and TGF-β signalling and tumour growth [17,61]. These findings raise a research question but cannot distinguish cause from tumour-related retention, inflammation, or altered tissue composition. Similarly, detection in kidney stones and supportive experimental lithogenic mechanisms [18,57] require prospective validation before MNPs can be considered a clinical stone risk factor.

No direct-particle study has yet established that a measured exposure precedes incident AKI, sustained eGFR decline, persistent albuminuria, a defined tubulopathy, CKD progression, or kidney failure. No exposure threshold or reference interval has been validated, and no assay has demonstrated clinical utility.

### 7.2. Plastic-Associated Chemicals Are Not PARTICLE Biomarkers

Large epidemiological studies of phthalates, bisphenols, and other plastic-associated chemicals are often discussed alongside MNPs. They are relevant to the plastics exposome and to particle–chemical mixtures, but urinary metabolites measure soluble chemical exposure and metabolism, not the number or mass of intact particles. Renal function can also affect the concentration and creatinine normalization of urinary metabolites, creating reverse-causality and collider-bias concerns.

In 9008 NHANES participants, several phthalate metabolites and bisphenol A were associated with lower eGFR or higher albumin-to-creatinine ratio, with results influenced by the method used to adjust for urine dilution [62]. Among 9989 adults, MBzP, MCPP, and MEOHP were positively associated with low eGFR, whereas other metabolites showed inverse associations and no overall association with albuminuria was found [63]. In 1610 adolescents, a higher phthalate-mixture concentration was associated with higher eGFR, interpreted as possible hyperfiltration, but the authors explicitly acknowledged reverse causality [64]. Newer analyses of PFAS–phthalate mixtures [65] and urinary or circulating plasticizer metabolites [66] likewise report heterogeneous associations.

This epidemiology supports concern about plastic-associated chemicals and renal function. It cannot be used as confirmatory human evidence for particle toxicity. Combining direct MNP measurements and chemical-metabolite studies into one effect estimate would conflate different exposures, kinetics, and causal pathways.

### 7.3. What Can Currently Be Concluded from Human Data

The human evidence supports four graded conclusions. First, polymer-confirmed particles or polymer-associated signals have been reported in blood and kidney-related specimens, supporting systemic presence and compatibility with renal exposure, while anatomical compartment and parenchymal access remain unresolved. Second, urine can contain polymer-confirmed particles or polymer mass, but its relation to systemic dose and renal clearance is unresolved. Third, preliminary associations with low-range albumin excretion, fetal renal biomarkers, renal tumours, or stones warrant replication but do not establish causal renal disease. Fourth, no direct study currently supports patient-level diagnosis, prognosis, or treatment based on an MNP measurement. The design, methods, limitations, and qualitative evidence level of the principal direct human and dialysis studies are summarized in Table 3.

## 8. Susceptible Populations and Bidirectional Risk

### 8.1. Chronic Kidney Disease

CKD may increase both exposure-related vulnerability and uncertainty in biomonitoring. Reduced nephron number and renal reserve, endothelial dysfunction, oxidative stress, chronic inflammation, impaired mitochondrial function, and maladaptive repair could magnify a particle-associated insult. Conversely, reduced filtration, altered protein binding, tubular dysfunction, or changes in urine concentration could modify blood or urinary MNP measurements independently of exposure. A higher circulating level might therefore reflect greater intake, reduced clearance, redistribution, or analytical differences; a lower urinary level might indicate lower exposure, lower filtration, or greater retention.

Experimental evidence supports susceptibility but not its magnitude in patients. In adenine-induced CKD, polystyrene exposure further reduced creatinine clearance and increased plasma urea and creatinine, albuminuria, tubular injury, inflammation, oxidative stress, apoptosis, and interstitial fibrosis; effects were associated with NF-κB and ERK/p38 activation and reduced sirtuin-1 [69]. In db/db mice, polystyrene exposure worsened biochemical injury, inflammation, inflammasome activation, and fibrosis [70]. Prior exposure also aggravated renal ischaemia–reperfusion injury and NLRP3–gasdermin-mediated pyroptosis [58]. These models show effect modification by a diseased kidney, but the exposure route and dose, the animal disease model, and the predominance of polystyrene limit quantitative translation.

Prospective human studies should treat kidney function as a time-varying determinant of both measured exposure and outcome. Baseline and repeated eGFR, measured GFR in nested studies, albuminuria, tubular injury markers, urine flow, medication use, diet, socioeconomic factors, and co-pollutants should be incorporated. Analyses that simply adjust a spot urinary particle concentration for creatinine risk introducing rather than removing bias.

### 8.2. Development, Pregnancy, and Early Life

Nephron formation is completed before birth, so developmental disruption may have lifelong consequences even without an immediate change in serum creatinine. Kidney organoids consistently show altered nephron-progenitor survival, tubular patterning, and energy metabolism after polystyrene exposure [40,41,42,43,44]. Maternal exposure in mice produced sex-specific reductions in glomerular number, immune and apoptotic responses, and fibrosis in offspring [71]. Human placental measurements associated with cord-blood filtration markers [60] provide an epidemiological signal, but they do not yet connect exposure with nephron endowment, childhood blood pressure, albuminuria, or longitudinal GFR.

Research in pregnancy and children requires especially stringent contamination control because collection devices, diapers, specimen bags, and clinical tubing may introduce plastics. It also requires age-appropriate outcomes and long follow-up. The detection of urinary MNPs in children [26] should motivate standardized exposure studies, not screening or attribution of developmental symptoms to MNPs.

### 8.3. Nephrolithiasis, Renal Malignancy, and Transplantation

Kidney stones, renal tumours, and transplanted kidneys are emerging but preliminary contexts. Stone matrices could archive long-term contact and experimental data support interactions with calcium-oxalate crystallization [18,57]. Tumour tissues may retain a different polymer profile and experimental exposure can modify oncogenic signalling [17,61]. Donor kidneys and associated tissues can contain polymer signals before implantation [16]. In each setting, temporal direction is unresolved and tissue architecture may determine particle retention. These studies should be viewed as starting points for disease-specific cohorts rather than evidence for clinical testing.

## 9. Dialysis and Healthcare-Associated Exposure

Dialysis creates a distinctive exposure environment because treatment fluids and blood encounter large surface areas of polymeric tubing, bags, filters, connectors, and membranes. Haemodialysis (HD) additionally uses hundreds of litres of treated water per week, while peritoneal dialysis (PD) involves repeated storage, warming, and transfer of sterile fluid through plastic systems. These features make dialysis a priority setting for MNP research [72]. They do not establish that particles cross the dialyser or peritoneum, accumulate in the patient, or cause outcomes.

The first direct comparison of dialysis fluids analysed 30 HD and PD solution samples and reported 36 suspected particles, predominantly fibres, with polyethylene, polyvinyl chloride, and ethylene-vinyl acetate among the identified polymers [67]. Concentrations were similar in HD and PD fluids (approximately 0.29 and 0.34 particles/L), but estimated weekly particle contact was higher for PD because of the larger sampled volume assumption. The particles were relatively large—mean size approximately 1.31 mm in HD and 0.64 mm in PD—making systemic passage uncertain. The study established possible contamination of treatment fluids, not the delivered patient dose.

A subsequent dual-simulation clinical study measured water before and after passage through HD equipment and blood before and after a session in nine patients [68]. No particles were detected in pre-dialysis water samples, whereas four post-equipment samples contained particles, mostly 20–50 µm. Median blood counts increased from 46.11 particles/mL before HD to 96.11 particles/mL after HD (*p* = 0.027), and several polymers appeared only after treatment. The paired design is informative, but the very small sample, absence of a full flow-normalized mass balance, possible haemoconcentration, vascular-access and sampling contamination, and lack of clinical endpoints prevent inference about retained dose or harm.

PD introduces different questions. Particles may originate from bags, warming, connectors, or transfer sets; conversely, effluent may contain material removed from the peritoneal cavity, shed by mesothelial or inflammatory processes, or introduced during collection. A narrative analysis of dialysis effluent and possible remediation approaches underscores the need for source apportionment [73]. Metal–organic frameworks or other experimental removal systems are not ready for clinical application because adsorption efficiency, biocompatibility, leaching, sterility, and effects on dialysis performance must first be demonstrated.

Current dialysis-fluid standards specify chemical and microbiological quality but do not provide particle-specific limits for MNPs [74]. Adding a regulatory threshold would be premature without a validated sampling protocol, harmonized size range and unit, blank strategy, relation to patient dose, and evidence of health effects. The appropriate next step is a complete mass-balance design rather than isolated concentration measurements.

**Figure 3:**
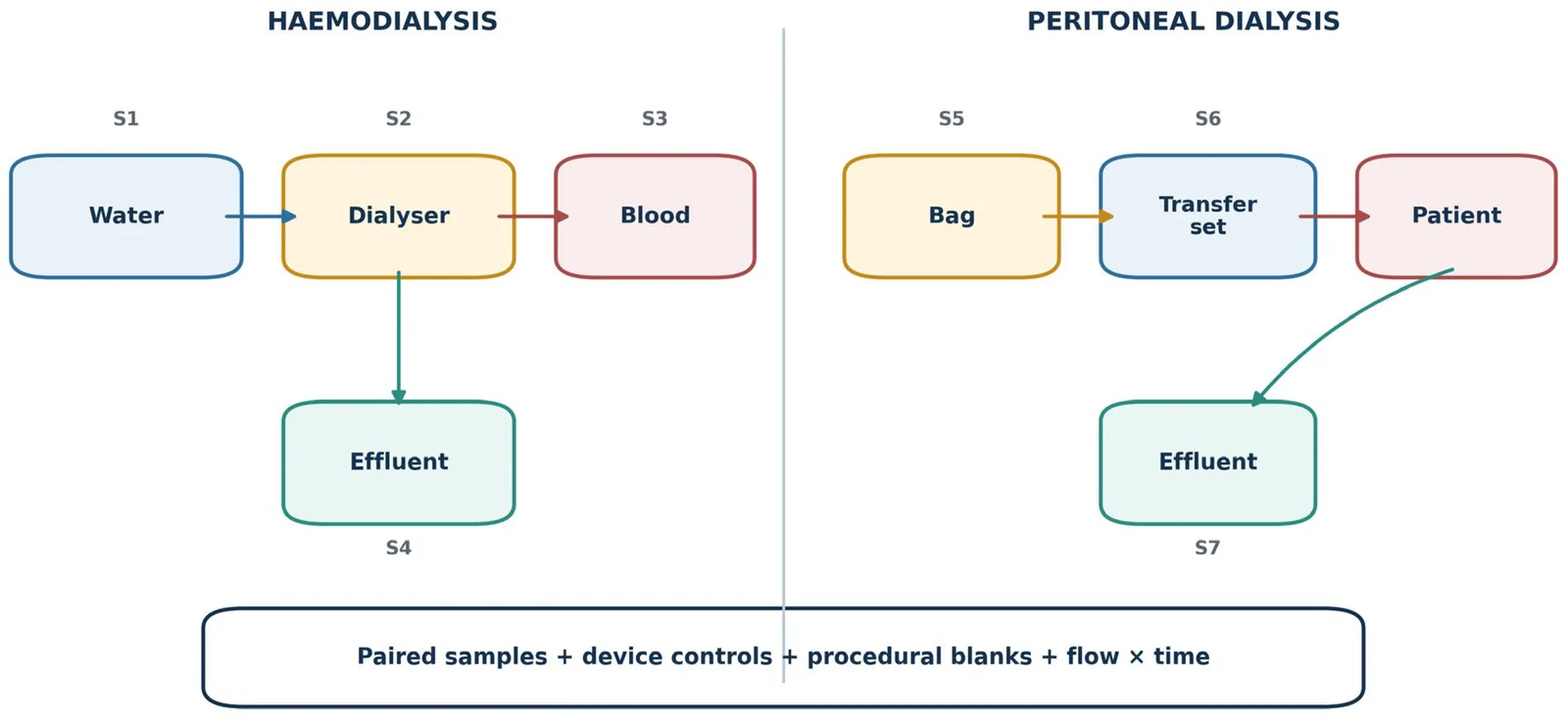
Dialysis as a particle mass-balance problem. In haemodialysis, source water, concentrate, dialysate, tubing, membrane, patient blood, and spent dialysate are potential sources or sinks. In peritoneal dialysis, the bag, warming, transfer set, peritoneal cavity, and drained effluent must be considered. Paired inflow/outflow and pre/post-patient measurements, normalized to volume and time and accompanied by device controls and procedural blanks, are required to estimate net patient exposure.

For HD, samples should be obtained from source water, post-treatment water, concentrate, freshly prepared dialysate, dialyser inlet and outlet, spent dialysate, and patient blood immediately before and after treatment. Blood volume, ultrafiltration, haematocrit, treatment time, membrane, tubing lot, and access type should be recorded. For PD, unopened bag fluid, post-warming fluid, transfer-set effluent, and timed drained dialysate should be compared. In both modalities, device-only circulation controls and full procedural blanks are essential. Only a flow- and time-integrated balance can distinguish introduction, adsorption to equipment, redistribution, and removal. The corresponding mass-balance framework is illustrated in Figure 3.

## 10. Clinical Implications and Research Priorities

### 10.1. What Clinicians Should and Should Not Do

The current evidence does not support routine measurement of MNPs in blood, urine, kidney tissue, dialysis fluid, or dialysate for individual clinical care. There is no validated reference interval, disease threshold, prognostic model, or treatment decision linked to a result. A positive research assay cannot identify the source, duration, retained renal dose, or attributable fraction of a patient’s kidney disease. Commercial “microplastic testing” or “detoxification” should not be recommended on the basis of the present renal literature.

No pharmacological intervention has clinical evidence for MNP-associated kidney injury. Antioxidants, ferroptosis inhibitors, complement blockade, sirtuin or PGC1α modulation, curcumin, and other agents have attenuated selected experimental pathways, but these findings identify mechanisms rather than therapies. Extrapolating doses or agents from animals and organoids would expose patients to uncertain benefit and potential harm.

Likewise, current data do not justify changing dialysis modality, membrane, prescription, or access. The available dialysis studies are designed for detection and source discovery, not comparative effectiveness or safety. Dialysis units can nevertheless support research-ready good practice by documenting tubing, membranes, bags, filters, and lots; minimizing unnecessary plastic contact where validated alternatives exist; and preserving samples during product investigations. Any material substitution must maintain sterility, biocompatibility, solute clearance, and mechanical safety.

General measures that reduce avoidable plastic use or unnecessary heating of plastic food-contact materials may be environmentally prudent, but no renal outcome trial has established a kidney-specific benefit.

### 10.2. Priorities for Translational Research

The next generation of studies should connect exposure, internal dose, renal compartment, phenotype, and time. Analytical studies should use harmonized collection and blank procedures, orthogonal confirmation, standardized reporting of number and mass, and explicit size windows. Kinetic studies should obtain paired blood and timed urine, quantify flow and renal function, and distinguish intact particles from polymer-associated chemical signals. Where kidney tissue is clinically available, spatially resolved methods should preserve glomerular, tubular, vascular, and interstitial localization.

Experimental designs should compare polymers beyond polystyrene, irregular and aged particles, environmentally plausible mixtures, chronic low-dose exposure, recovery, and pre-existing kidney disease. Reporting nominal concentration is insufficient; studies should quantify the administered, systemic, and renal delivered dose wherever possible. Organoids and microphysiological kidney systems would benefit from perfusion, immune and endothelial components, and measurement of transepithelial transport.

Prospective human cohorts should prespecify incident AKI, sustained eGFR decline, persistent albuminuria, tubular dysfunction, nephrolithiasis, CKD progression, and kidney failure rather than combine nonspecific biomarkers. Repeated direct MNP measures should precede outcomes, and models must account for kidney function as both a confounder and possible mediator. Biobanks linked to renal phenotyping could enable nested multimodal studies without exposing patients to research biopsies.

Dialysis research should progress from detection to source apportionment, mass balance, reproducibility across equipment and lots, and patient outcomes. A multicentre study should pair device-only circuits with patient sessions, correct blood concentrations for plasma-volume change, integrate flow and treatment duration, and sample both blood and effluent. Only then can mitigation strategies be evaluated. Priority studies needed to establish clinical relevance are summarized in Table 4.

## 11. Conclusions

MNPs have been detected in human blood, urine, and kidney-related tissues, and the experimental literature provides a coherent account of how selected particles can injure renal systems. Mitochondrial and ER stress, disrupted autophagy and lysosomal handling, innate immune activation, regulated cell death, tubular senescence, and fibrosis recur across cell, organoid, and animal models. The strength of this evidence is biological plausibility, not quantitative human risk.

The unresolved step is translation from experimental hazard to human disease. Direct human studies remain few, largely cross-sectional or exploratory, and unable to establish temporality, anatomical dose, or renal dose–response. CKD and dialysis further complicate interpretation by modifying susceptibility, measured concentrations, and potential exposure pathways.

MNP-associated renal injury should therefore be regarded as a credible emerging toxicological concern rather than a current clinical diagnosis. Progress requires standardized multimodal analysis, paired-matrix toxicokinetics, realistic dose-anchored models, prospective renal cohorts, and complete dialysis mass-balance studies; patient-level testing, treatment, or dialysis changes remain unsupported.

## Figures and Tables

**Table 1:** Principal analytical approaches and their interpretation in renal research.

Approach	Primary Output	Main Strength	Main Limitation	Minimum Renal-Study Safeguards
Raman or micro-Raman spectroscopy	Particle count, size, shape, polymer spectrum	Particle-specific chemical identification; morphology retained	Fluorescence, spectral ambiguity, low throughput, limited nanoscale detection	Procedural blanks; library and match threshold; recovery; proportion of suspected particles confirmed
µFTIR or LD-IR spectroscopy	Particle count, size, shape, polymer spectrum	Rapid mapping of micrometre-scale particles	Size-dependent sensitivity; substrate and matrix effects	Blank correction; size window; substrate controls; manual validation of automated classification
Py-GC/MS	Polymer mass	Sensitive multi-polymer quantification	Destructive; no morphology or anatomical localization; particulate state may be ambiguous	Matrix-matched calibration; separation definition; polymer-specific limits; blank subtraction
Electron microscopy with elemental analysis	Ultrastructure and elemental composition	Nanoscale morphology and localization	Polymer identity often indirect; small field of view	Correlative spectroscopy; representative sampling; contamination controls
Fluorescence-labelled particles	Experimental kinetics and cellular uptake	High temporal and spatial sensitivity	Dye leaching, photobleaching, autofluorescence, label–particle dissociation	Free-dye and dialysate controls; orthogonal confirmation of intact particles
Multimodal analysis	Complementary particle and mass information	Reduces platform-specific blind spots	Complex, expensive, and still dependent on harmonized sampling	Prespecified concordance rules; shared blanks; transparent handling of discordant results

**Table 2:** Representative experimental evidence for MNP-associated renal injury.

Model and Exposure	Principal Renal Finding	Mechanistic Support	Main Translational Limitation
HK-2 cells; polystyrene MPs/NPs [36,37]	Mitochondrial ROS, ER stress, autophagy, MAPK activation, apoptosis	MitoTEMPO attenuation; pathway coherence	Direct nominal exposure; immortalized monolayer; high concentrations
HK-2 cells; PS, PMMA, and PE NPs [38]	Polymer-, size-, and concentration-dependent uptake and cytotoxicity	Cross-material comparison and dose response	Short exposure; no biological ageing or clearance
Proximal tubular cells; PE-MPs plus BPA [39]	Greater oxidative, inflammatory, and viability effects with co-exposure	Comparison with single exposures	Does not separate particle from chemical contribution in vivo
Human kidney organoids [40,41,42,43,44]	Reduced growth, abnormal nephron patterning, metabolic rewiring, autophagy, apoptosis	DDIT4 silencing; convergent structural and omics findings	Developmental immaturity; no perfusion, filtration, or excretion
Repeated-dose mice; PS particles [36,45,46,47,48,49,50,51,54,55]	Tubular/glomerular injury, inflammation, senescence, ferroptosis, fibrosis	Complement, NLRP3, Klotho, ferroptosis, lysosome, PGC1α, and TGF-β interventions	Predominantly pristine PS; administered dose not anchored to human renal dose
Polyethylene rodent models [52,53]	Imaging, biochemical, mitochondrial, glomerular, and tubular dysfunction	Dose response and organ-function measures	High oral dose; uncertain environmental comparability
Irregular versus spherical particles [35]	Shape-dependent circulation, glomerular lesions, and albumin hyperfiltration	Matched-shape comparison; intravital and isolated-kidney methods	Short experimental exposure; label and species effects
Degraded versus polymeric PLA [56]	Greater macrophage uptake and renal inflammation with oligomeric material	MSR1 and PI3K/AKT inhibition	Model degradation state; human exposure unknown
CaOx crystal and nephrolithiasis models [57]	Increased crystal adhesion, ferroptosis, inflammation, and stone formation	LRP6 manipulation and in-vivo confirmation	Co-exposure regimen; human temporal relation unknown

**Table 3:** Design, methods, limitations, and qualitative evidence level of direct human and dialysis studies relevant to renal health. Evidence level describes the strength of clinical inference, not analytical detection alone.

Study Design, Participants, and Matrix	Analytical Method	Main Renal Observation	Principal Limitations	Qualitative Evidence Level
Cross-sectional detection; 10 nephrectomy kidneys and 10 unpaired urine donors [7]	Micro-Raman	Polymer-confirmed particles in kidney and urine specimens	Small, unpaired samples; digestion removed localization; no phenotype	Direct detection; very low clinical certainty
Exploratory donor–recipient study; 28 donors; kidney, renal vessels, ureter, and adrenal tissue [16]	Py-GC/MS, LD-IR, electron microscopy	Tissue polymer signals; exploratory early post-transplant blood-pressure correlations	Multiple tissues and tests; localization unresolved; no robust corrected association	Direct detection plus exploratory association; very low certainty
Paired tumour study; 32 ccRCC patients [17]; mechanistic tissue/model study including 3 patients [61]	Py-GC/MS, LD-IR, electron microscopy; experimental models	Higher tumour-associated polymer signals and pathway activation	Reverse causality, tumour-related retention, small mechanistic tissue series	Hypothesis-generating; very low clinical certainty
Cross-sectional stone study; 10 patients; kidney stones plus CaOx models [18,57]	Multimodal polymer analysis; mechanistic cell and animal models	Polymers in stones; experimental promotion of CaOx-associated injury	Temporal sequence absent; incorporation and contamination unresolved	Hypothesis-generating; very low clinical certainty
Cross-sectional paired-matrix study; 98 environmentally exposed adults; blood and urine [25]	Py-GC/MS with multivariate analysis	Polymer-specific blood–urine partitioning patterns	No timed clearance, flow normalization, or renal outcome	Direct detection; no renal-risk inference
Cross-sectional study; 29 schoolchildren; first-morning urine [26]	SEM/EDX with Py-GC/MS characterization	MNP signals reported in all urine samples	Single urine sample; collection, source, and clearance unresolved	Direct detection; no renal-risk inference
Cross-sectional study; 50 healthy adults; spot urine and stool [59]	HPLC-HRMS	Any urinary polymer associated with higher low-range uACR	Multiple testing; all uACR <30 mg/g; no temporality	Preliminary association; very low certainty
Birth cohort; 1350 pregnancies; placenta and cord-blood renal biomarkers [60]	Placental particle-count analysis	Cord creatinine and cystatin-C mixture associations	Placenta is an exposure proxy; perinatal determinants; no renal follow-up	Observational association; low certainty
Analytical cross-sectional study; 30 HD/PD treatment-fluid samples [67]	FTIR	Suspected particles in treatment fluids	Large particles; limited samples; patient transfer not measured	Source detection; no patient-dose inference
Paired pre/post clinical study plus device simulation; 9 HD patients; water and blood [68]	LD-IR	Higher post-HD blood counts (particles/mL)	Very small study; no flow-normalized mass balance or clinical outcome	Session-associated signal; very low certainty

**Table 4:** Research agenda needed to establish clinical relevance.

Question	Minimum Design	Critical Measurements	Decision Enabled
What reaches the human kidney?	Paired blood–urine kinetics with clinically available tissue	Polymer, size, shape, mass, count, blanks, recovery, renal localization	Valid internal and renal dose metric
Are urinary MNPs a renal biomarker?	Repeated timed urine across kidney-function strata	Urine flow, paired blood, eGFR/measured GFR, tubular markers, within-person variability	Interpretation of urinary concentrations
Do MNPs cause kidney disease?	Prospective cohort with repeated direct exposure measurement	Incident AKI/CKD, sustained eGFR slope, persistent albuminuria, co-exposures, causal modelling	Human risk estimate and threshold
Which particle properties matter?	Factorial chronic models with realistic particles	Polymer, size, shape, ageing, corona, degradation, delivered renal dose	Hazard ranking and material design
Who is susceptible?	CKD, diabetes, pregnancy, childhood, and older-adult cohorts	Baseline disease, medication, inflammation, renal reserve, longitudinal outcomes	Risk stratification
Does dialysis add or remove MNPs?	Multicentre full mass-balance studies	Device-only controls, inflow/outflow, pre/post blood, effluent, flow, time, haemoconcentration	Source control and comparative equipment evaluation
Can exposure be reduced safely?	Controlled engineering or clinical intervention	Particle reduction, dialysis/clinical performance, adverse effects, renal outcomes	Evidence-based mitigation

## Data Availability

No new data were created or analysed in this study.

## Abbreviations

AKI	Acute kidney injury
BPA	Bisphenol A
CaOx	Calcium oxalate
ccRCC	Clear-cell renal cell carcinoma
CKD	Chronic kidney disease
DEHP	Di(2-ethylhexyl) phthalate
eGFR	Estimated glomerular filtration rate
ER	Endoplasmic reticulum
FTIR	Fourier-transform infrared spectroscopy
HD	Haemodialysis
HPLC-HRMS	High-performance liquid chromatography coupled to high-resolution mass spectrometry
LD-IR	Laser direct infrared spectroscopy
MNPs	Micro- and nanoplastics
MPs	Microplastics
NPs	Nanoplastics
PD	Peritoneal dialysis
PE	Polyethylene
PMMA	Poly(methyl methacrylate)
PS	Polystyrene
Py-GC/MS	Pyrolysis gas chromatography–mass spectrometry
ROS	Reactive oxygen species
uACR	Urinary albumin-to-creatinine ratio
